# Submarine Outfalls of Treated Wastewater Effluents are Sources of Extensively- and Multidrug-Resistant KPC- and OXA-48-Producing *Enterobacteriaceae* in Coastal Marine Environment

**DOI:** 10.3389/fmicb.2022.858821

**Published:** 2022-05-06

**Authors:** Marija Kvesić, Ivica Šamanić, Anita Novak, Željana Fredotović, Mia Dželalija, Juraj Kamenjarin, Ivana Goić Barišić, Marija Tonkić, Ana Maravić

**Affiliations:** ^1^Center of Excellence for Science and Technology, Integration of Mediterranean Region, University of Split, Split, Croatia; ^2^Doctoral Study of Biophysics, Faculty of Science, University of Split, Split, Croatia; ^3^Department of Biology, Faculty of Science, University of Split, Split, Croatia; ^4^School of Medicine, University of Split, Split, Croatia; ^5^University Hospital Split, Split, Croatia

**Keywords:** carbapenemase-producing *Enterobacteriaceae*, marine environment, coastal waters, Croatia, KPC, OXA-48

## Abstract

The rapid and ongoing spread of carbapenemase-producing *Enterobacteriaceae* has led to a global health threat. However, a limited number of studies have addressed this problem in the marine environment. We investigated their emergence in the coastal waters of the central Adriatic Sea (Croatia), which are recipients of submarine effluents from two wastewater treatment plants. Fifteen KPC-producing *Enterobacteriaceae* (nine *Escherichia coli*, four *Klebsiella pneumoniae* and two *Citrobacter freundii*) were recovered, and susceptibility testing to 14 antimicrobials from 10 classes showed that four isolates were extensively drug resistant (XDR) and two were resistant to colistin. After ERIC and BOX-PCR typing, eight isolates were selected for whole genome sequencing. The *E. coli* isolates belonged to serotype O21:H27 and sequence type (ST) 2795, while *K. pneumoniae* isolates were assigned to STs 37 and 534. Large-scale genome analysis revealed an arsenal of 137 genes conferring resistance to 19 antimicrobial drug classes, 35 genes associated with virulence, and 20 plasmid replicons. The isolates simultaneously carried 43–90 genes encoding for antibiotic resistance, while four isolates co-harbored carbapenemase genes *bla*_KPC-2_ and *bla*_OXA-48_. The *bla*_OXA-48_ was associated with IncL-type plasmids in *E. coli* and *K. pneumoniae*. Importantly, the *bla*_KPC-2_ in four *E. coli* isolates was located on ~40 kb IncP6 broad-host-range plasmids which recently emerged as *bla*_KPC-2_ vesicles, providing first report of these *bla*_KPC-2_-bearing resistance plasmids circulating in *E. coli* in Europe. This study also represents the first evidence of XDR and potentially virulent strains of KPC-producing *E. coli* in coastal waters and the co-occurrence of *bla*_KPC-2_ and *bla*_OXA-48_ carbapenemase genes in this species. The leakage of these strains through submarine effluents into coastal waters is of concern, indicating a reservoir of this infectious threat in the marine environment.

## Introduction

Antibiotic resistance is one of the greatest threats to global health nowadays, leading to the higher mortality rates and increased economic costs (Pulingam et al., 2021). The natural environment has been recognized as one of its major reservoirs (Amarasiri et al., 2020), as antibiotic-resistant human pathogens have been detected in coastal marine areas (Šamanić et al., 2021), rivers (Ekwanzala et al., 2020), lakes (Su et al., 2020) and shellfish (Maravić et al., 2013). Effluents from the wastewater treatment plants (WWTPs) have been evidenced as particularly significant routes for dissemination of antibiotic resistance in the natural environment (Ekwanzala et al., 2020), especially the hospital effluents through which the emerging opportunistic pathogens directly enter from the hospital to the aquatic environment (Ekwanzala et al., 2019).

In recent decades, the rapid spread of Gram-negative bacteria resistant to the most potent β-lactam antibiotics, the carbapenems, and the continuous emergence of new resistant strains have raised the global alarm. In 2017, the World Health Organization defined priority categories for emerging multidrug-resistant pathogens for which new antimicrobials are urgently needed, with carbapenem-resistant *Enterobacteriaceae* (CRE) identified as critical (WHO, 2017). Carbapenem resistance in these bacteria arises mainly from the production of carbapenemases, of which KPC, SME, IMI and NMC belonging to Ambler class A, IMP, VIM and NDM metallo-β-lactamases (MBLs) belonging to class B, and OXA-48 and its derivatives belonging to class D have been detected worldwide (Bonomo et al., 2018; Brolund et al., 2019). In Croatia, CRE isolates are being increasingly reported in hospitals, mainly due to the rapid spread of KPC-producing *Enterobacteriaceae* first in the northwest (Jelić et al., 2016) and later in the southern coastal regions (Bedenić et al., 2021).

Considering the importance of CRE for public health and the One Health approach, we aimed to study for the first time their occurrence in the coastal waters of the eastern Adriatic, focusing on the area influenced by the submarine sewage outlets of two WWTPs, which could serve as potential routes for the introduction of these bacteria into the coastal marine environment. The isolated CRE were analyzed by PCR for the presence of carbapenemase-encoding genes, after which eight KPC-2-producing *Enterobacteriaceae* isolates were subjected to high-throughput DNA sequencing. We then performed a detailed search of the obtained genome sequences, focusing on the antibiotic resistance genes (ARGs), virulence factors and plasmid replicons. The isolates were also assigned to sequence types (STs) using the multi-locus sequence typing (MLST) scheme and their serotype was determined. This study led to the first identification of potentially virulent CRE in the marine environment in Croatia, evidencing a transmission route through submarine outfalls and a new reservoir of these opportunistic pathogens in Croatia outside hospital settings.

## Materials and Methods

### Sampling

**Figure 1:**
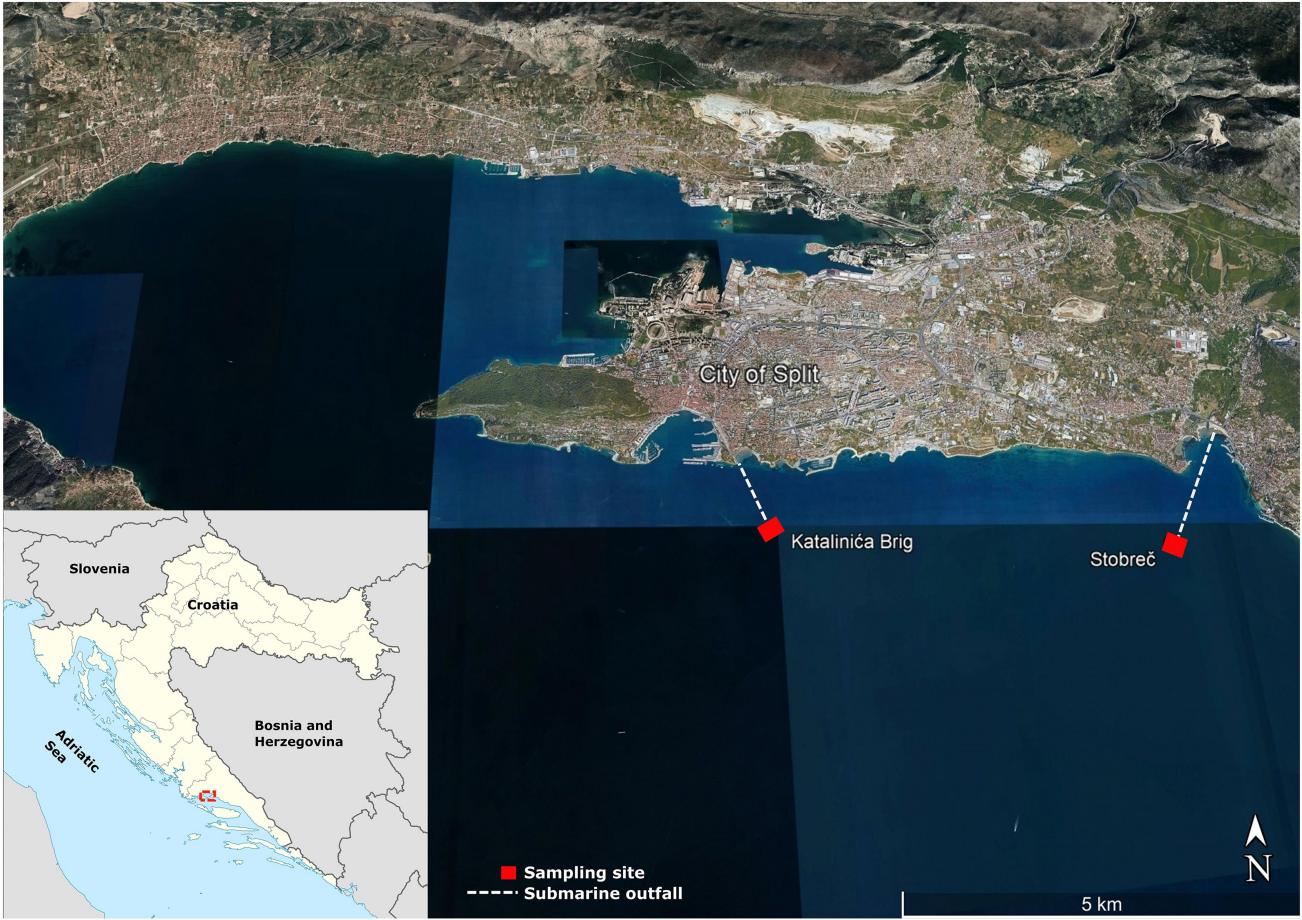
Sampling sites (represented by squares) at the submarine outfalls of the two WWTPs, central Adriatic Sea, Croatia.

Fifteen *Enterobacteriaceae* isolates were recovered in June, July, and September 2020 as part of the project aimed to study the impact of treated submarine effluents in the coastal waters of the central Adriatic Sea in Croatia. Details of the sampling procedure and locations have been described previously (Kvesić et al., 2021). Briefly, the study focused on submarine effluents from the two WWTPs, the Katalinića brig and the Stupe-Stobreč, which mechanically treat wastewater from the wider Split area at an average flow rate of 35,000 and 30,000 m^3^/day, respectively, and discharge it through submarine outfalls into the coastal waters of the Brač and Split channels (Figure 1). While the Stobreč WWTP processes only municipal wastewater, the Katalinića brig WWTP treats municipal wastewater and stormwater runoff. The Katalinića brig WWTP also collects wastewater from the University Hospital Centre Split, the largest medical center in southern Croatia with 1,400 beds serving a population of approximately 500,000, which increases sharply in the summer months during the tourist season. The submarine outfalls of the Katalinića brig (43°29′22.7 ″N, 16°27′11.2 ″E) and the Stobreč WWTP (43°28′53.6 ″N 16°31′04.3 ″E) are located at a depth of 42 and 37 m, respectively.

Water samples were collected from the boat using a Niskin sampler, transferred to sterile 1 L bottles, protected from light, and transported to the laboratory for further analysis within 4 h at 4°C.

### Bacterial Identification and Antibiotic Susceptibility Testing

One hundred milliliters of the water samples were filtered through 0.2 μm pore size MCE membrane filters (GE Healthcare, United Kingdom), which were then placed on CHROMID® Carba agar (bioMérieux, France) and incubated for 48 h. This chromogenic medium selects for the growth of CRE and allows the typical pink to the burgundy appearance of *Escherichia coli* colonies and blue-green to blue-grey of *Klebsiella*, *Enterobacter*, *Serratia* and *Citrobacter* spp. Incubation was carried out at 42°C to suppress the growth of autochthonous environmental species that are unable to grow under mesophilic conditions. Based on colony morphology, all putative Enterobacteriaceae isolates were cultivated in pure culture on MacConkey agar (Biolife, Italy) at 37°C for 18 h and identified to species level using MALDI-TOF MS (Microflex LT mass spectrometer and MALDI Biotyper 4.1.80, Bruker Daltonics, Germany).

The isolates were tested for susceptibility to 14 antibiotics using Etest strips (AB Biodisk, Sweden) except for colistin (CL) whose susceptibility was tested using the broth microdilution method. The tests were performed, and the minimum inhibitory concentrations (MICs) were interpreted based on the European Committee on Antimicrobial Susceptibility Testing (EUCAST) guidelines (EUCAST, 2020). The MIC value of CL was recorded as the lowest concentration showing no visually detectable bacterial growth in the 96-well microtiter plates and was the consensus value of the experiment performed in triplicate. The antibiotics tested (except CL) and their maximum concentrations were as follows: piperacillin/tazobactam (TZP, 256 μg), piperacillin (PIP, 256 μg), ceftazidime (CAZ, 256 μg), cefotaxime (CTX, 32 μg), cefepime (FEP, 256 μg), aztreonam (ATM, 256 μg), imipenem (IMP, 32 μg), meropenem (MER, 32 μg), ertapenem (ETP, 32 μg), ciprofloxacin (CIP, 32 μg), gentamicin (GEN, 256 μg), tetracycline (TET, 256 μg), and trimethoprim-sulfamethoxazole (SXT, 1/19 μg). *E. coli* ATCC 25922 was used as a control. According to Magiorakos et al. (2012), multidrug-resistant (MDR) phenotype was defined as acquired non-susceptibility to at least one antibiotic from three or more classes, while the extensively drug-resistant (XDR) phenotype was designated as non-susceptibility to at least one agent from all but two or fewer antibiotic classes (i.e., remaining susceptible to only one or two categories).

Isolates were further tested for the presence of class C AmpC β-lactamases with AmpC Etest (AB Biodisk) and carbapenemases with MBL Etest (AB Biodisk) and Rapidec Carba NP test (bioMérieux) according to the manufacturer’s instructions. The production of extended-spectrum β-lactamases (ESBLs) was tested using clavulanic acid (CLA) combination discs. The phenotype consistent with the production of ESBLs was defined by an increase in zone diameter of ≥5 mm for CAZ and/or CTX in combination with CLA compared to its zone when tested alone (EUCAST, 2013).

### PCR Screening for Carbapenemase Encoding Genes and *mcr-1* Gene

Genomic DNA was extracted using the NucleoSpin Microbial DNA kit (Macherey-Nagel, United Kingdom) and the concentration and quality of DNA were analyzed using the NanoDrop® Spectrophotometer 1000 (Thermo Scientific, United States). Multiplex PCR assays were performed to screen for the presence of carbapenemase genes encoding class A KPC and class B IMP, VIM and NDM using the primers and PCR conditions described previously (Poirel et al., 2011). Isolates were screened by standard PCR for the presence of the *mcr-1* gene, which encodes plasmid-mediated colistin resistance (Liu et al., 2016). The amplified fragments were separated on a 1% (w/v) agarose gel, purified using the ReliaPrep™ DNA Clean-Up and Concentration System (Promega, United States) and subjected to Sanger sequencing of both strands in Macrogen Europe service (Netherlands). The obtained nucleotide sequences were compared with the homologous sequences from the GenBank database using the BLASTn algorithm.^1^

### Molecular Typing of Bacterial DNA

To exclude the possibility of clonal relatedness between the isolates of the same species, enterobacterial repetitive intergenic consensus (ERIC) and BOX PCR analyses were performed using the primers and conditions previously described (Araújo et al., 2014).

### High-Throughput DNA Sequencing and Computational Data Analysis

Genomic DNA was sent to Novogene (Cambridge, United Kingdom) for whole genome sequencing (WGS) and bioinformatics analysis of the raw sequencing data. DNA libraries were prepared using the NEBNext® DNA Library Prep Kit (Illumina, United States) and, after a quality check, were subjected to pair-end sequencing on the Illumina NovaSeq 6000 platform with a read length of 150 bp at each end. The obtained reads were subjected to further quality control. Then, the clean reads were mapped to the reference genomes to detect and annotate single nucleotide polymorphism (SNP), structural variants (SV) and copy number variation (CNV) according to the mapping results. FASTQ files containing clean sequences were further analyzed using tools available at the Center for Genomic Epidemiology,^2^ including multi-locus sequence typing (MLST) with MLST 2.0, sequence type (ST) with SerotypeFinder 2.0, presence of virulence genes (VirulenceFinder 2.0), resistance genes (ResFinder 4.1), and plasmid replicons (PlasmidFinder 2.1). In addition, antibiotic resistance and virulence profiling was performed using the ARESdb cloud platform introduced by Ferreira et al. (2020).^3^ This involved searching for marker sequences with coverage of ≥60% and identity of ≥90% to those cataloged in ARESdb (Ferreira et al., 2021).

To link carbapenemase KPC-2 and OXA-48 encoding genes to specific Inc. plasmid groups, plasmids were reconstructed with SPAdes v3.13.1 from trimmed (trimmomatic v0.39) sequencing reads, after which the replicon types and resistance markers were cross referenced to the *de novo* assemblies (Kudirkiene et al., 2018). Plasmid Finder v2.1 determined replicon types from the WGS assemblies generated by SPAdes. Because plasmid reconstruction from short-read sequencing is challenging and assembly typically results in many fragmented contigs per genome of unclear origin, plasmidSPAdes tool was used to identify as much as plasmid contigs. The algorithm in plasmidSPAdes predicted which contigs belong to plasmid DNA and assigned those contigs into components. Components containing specific plasmid replicons and their combinations from a selected strain were further used to search against NCBI nr database using BlastN for the most similar plasmids. *De novo* assembled plasmids were aligned against ARESdb with thresholds set at >60% query coverage and > 90% alignment identity to detect resistance markers. Further analysis of the generated assemblies was conducted using the Proksee server to create circular alignments of the reads to the reference plasmids available in the NCBI database.^4^

## Results

This study investigated the emergence and antibiotic resistance of CRE in submarine effluent-receiving coastal waters of central Adriatic to contribute to the global surveillance of these opportunistic pathogens outside of hospital settings.

### Strain Isolation and Antibiotic Susceptibility Pattern

Twenty-two isolates that exhibited characteristic pink or blue-green colony morphology were recovered on selective CHROMID® Carba agar (bioMérieux). By MALDI-TOF MS, seven isolates were identified as *Enterococcus faecium* and excluded from further investigation. The remaining 15 isolates belonged to the *Enterobacteriaceae* family (nine *E. coli*, four *Klebsiella pneumoniae* and two *Citrobacter freundii*) and were obtained from water samples collected in June, July, and September 2020 near the submarine outfall of the Katalinića Brig WWTP. All but one isolate showed resistance to at least one carbapenem antibiotic. Among them, four isolates (one *E. coli* and three *K. pneumoniae*) were designated extensively drug resistant (XDR), while nine isolates (seven *E. coli*, one *K. pneumoniae* and one *C. freundii*) were multidrug resistant (MDR). Although two *K. pneumoniae* isolates (C1 and C2) were resistant to colistin, the *mcr-1* gene was not detected by PCR. The detailed antibiotic resistance profiles of the *Enterobacteriaceae* isolates are shown in Table 1.

**Table 1:** Antibiotic resistance profiles of 15 KPC-producing *Enterobacteriaceae* isolates recovered in this study^a^.

Isolate no.	M18	M12	M13	M14	M15	M16	M17	M19	M20	5a	M11	C2	C1	CF1	CF2
Species	*Escherichia coli*	*Escherichia coli*	*Escherichia coli*	*Escherichia coli*	*Escherichia coli*	*Escherichia coli*	*Escherichia coli*	*Escherichia coli*	*Escherichia coli*	*Klebsiella pneumoniae*	*Klebsiella pneumoniae*	*Klebsiella pneumoniae*	*Klebsiella pneumoniae*	*Citrobacter freundii*	*Citrobacter freundii*
Isolation date^b^	06/2020	06/2020	06/2020	06/2020	06/2020	06/2020	06/2020	06/2020	06/2020	06/2020	06/2020	07/2020	07/2020	09/2020	09/2020
KPC type	KPC-2	KPC-2	KPC-2	KPC-2	KPC-2	KPC-2	KPC-2	KPC-2	KPC-2	KPC-2	KPC-2	KPC-2	KPC-2	KPC-2	KPC-29
PIP	1.5	>256	>256	>256	>256	>256	>256	>256	>256	>256	>256	>256	>256	64	2
PIP/TZB	0.75	32	>256	>256	256	192	>256	>256	256	32	32	192	>256	8	1
CTX	0.047	16	16	>32	>32	>32	12	24	16	>32	>32	24	>32	32	0.5
CAZ	0.19	8	8	8	8	3	8	4	12	12	8	8	64	32	0.38
FEP	0.032	1.5	32	48	256	4	32	8	8	32	16	32	256	1.5	0.094
ATM	0.094	1.5	96	>256	>256	64	256	6	128	8	12	1,5	>256	6	0.125
IPM	0.19	>32	32	>32	24	8	32	8	8	8	8	>32	>32	0.19	0.19
MER	8	>32	>32	>32	>32	12	8	16	>32	32	6	>32	32	1	0.5
ETP	0.004	32	32	>32	8	8	32	24	32	>32	32	>32	>32	0.006	0.004
CIP	0.012	6	4	4	0.75	12	6	4	12	6	6	4	6	0.75	0.012
GEN	0.5	16	1	1	0.5	2	1	2	3	8	8	8	1	0.5	0.5
TET	4	16	2	3	32	3	3	2	12	12	>256	16	4	4	4
SXT	0.064	0.5	32	>32	0.75	0.19	>32	0.38	32	0.25	0.25	0.5	0.038	0.064	0.038
CL	0.125	2	0.125	0.25	0.125	0.5	0.125	0.0625	1	0.125	0.0625	32	16	0.125	0.0625
Resistance phenotype		MDR	MDR	MDR	MDR	MDR	MDR	MDR	XDR	XDR	XDR	XDR	MDR	MDR	
AmpC Etest	neg	pos	neg	neg	neg	neg	neg	neg	neg	neg	neg	neg	neg	pos	neg
Rapidec Carba NP test	pos	pos	pos	pos	pos	pos	pos	pos	pos	pos	pos	pos	pos	pos	pos
ESBL test	neg	pos	neg	pos	pos	neg	neg	neg	pos	pos	pos	pos	pos	neg	neg

TZP, piperacillin/tazobactam; PIP, piperacillin; CAZ, ceftazidime; CTX, cefotaxime; FEP, cefepime; ATM, aztreonam; IMP, imipenem; MER, meropenem; ETP, ertapenem; CIP, ciprofloxacin; GEN, gentamicin; TET, tetracycline; SXT, trimethoprim-sulfamethoxazole; CL, colistin; MDR, multidrug-resistant; XDR, extensively drug-resistant; neg, negative; and pos, positive.

a Resistance phenotype is indicated by shading according to EUCAST (2020) except for TET that was evaluated based on CLSI (2020) breakpoints.

b Isolation date is given as month/year.

The Rapidec Carba NP test indicated carbapenemase production in all 15 isolates. PCR screening and Sanger sequencing further confirmed the presence of the carbapenemase gene *bla*_KPC-2_ in all but one *C. freundii* isolate (CF2), which carried *bla*_KPC-29_. This carbapenemase gene is derived from the ancestral allele *bla*_KPC-3_, and its expression does not affect the activity of carbapenems (Hobson et al., 2020). However, the CF2 isolate remained sensitive to all beta-lactam antibiotics tested, including cephalosporins (Table 1), casting doubt on the full expression of this gene in this isolate.

Based on the ERIC and BOX profiles (Supplemental Figure S1), eight isolates (four *E. coli*, three *K. pneumoniae*, and one *C. freundii*) that exhibited the most diverse profiles were subjected to WGS.

The draft genome sizes of the isolates ranged from 5.1 to 5.8 Mb, with diverse sizes of N50, and numbers of coding sequences and contigs (Table 2). A total of 137 genes were identified mediating intrinsic or acquired resistance to 19 antimicrobial drug classes, including penicillins, cephamycins, cephalosporins, carbapenems, penems, monobactams, fluoroquinolones, aminoglycosides, macrolides, phenicols, quinolones, sulfonamides, trimethoprim, rifampicin, tetracyclines, fosfomycin, nitroimidazoles, peptides, and aminocoumarin (Supplemental Table S1). The isolates were found to harbor between 43 and 90 gene markers associated with resistance phenotypes, with the highest number detected in *E. coli* genomes (Table 3). Most of these genes were associated with intrinsic resistance mechanisms such as regulation and transport by resistance-nodulation-cell division (RND) and major facilitator superfamily (MFS) antibiotic efflux pumps or porin uptake (Supplemental Table S1). Moreover, the isolates harbored three to nine *bla* genes, out of which four isolates (*E. coli* M12, M14, and M20 and *K. pneumoniae* C2) simultaneously possessed two carbapenemase-encoding genes, *bla*_KPC-2_ and *bla*_OXA-48_. In addition, eight sequenced genomes possessed a total of 20 resistance-associated plasmid replicons and 35 genes involved in bacterial virulence (Table 3; Supplemental Table S1).

**Table 2:** Metadata of the whole-genome sequenced CRE isolates from Croatia.

	M12	M14	M17	M20	5a	M11	C2	CF1
Species	*Escherichia coli*	*Escherichia coli*	*Escherichia coli*	*Escherichia coli*	*Klebsiella pneumoniae*	*Klebsiella pneumoniae*	*Klebsiella pneumoniae*	*Citrobacter freundii*
Genome size (bp)	5,207,851	5,243,436	5,173,484	5,186,809	5,667,286	5,659,465	5,826,947	5,103,429
No. of CDS^a^	5,028	5,082	4,969	5,023	5,339	5,345	5,554	4,865
No. of contings	240	231	207	224	342	289	195	74
No. of contings >1,000 bp	159	164	135	164	204	199	132	45
Average depth (x)	223	185	205	199	224	248	229	264
GC content (%)	50.54	50.55	50.59	50.6	56.92	56.92	57.04	51.84
*N*_50_ (bp)	89,262	87,097	90,500	78,173	70,753	74,345	116,900	372,763
No. of tRNAs	78	81	79	82	77	77	81	78
SRA accession no.	SAMN22028927	SAMN22028930	SAMN22028929	SAMN22028928	SAMN22028932	SAMN22028933	SAMN22028931	SAMN22028934

a CDS, coding DNA sequences.

**Table 3:** Molecular characteristics of eight whole-genome sequenced CRE isolates.

	M12	M14	M17	M20	5a	M11	C2	CF1
Species	*Escherichia coli*	*Escherichia coli*	*Escherichia coli*	*Escherichia coli*	*Klebsiella pneumoniae*	*Klebsiella pneumoniae*	*Klebsiella pneumoniae*	*Citrobacter freundii*
Serotype	O21:H27	O21:H27	O21:H27	O21:H27				
ST	2795	2795	2795	2795	37	37	534	128
Selected antibiotic resistance genes (total no.)^a^	*dfrA14*, *qnrVC4*, *cmlA5*, *mdf(A)*, *mph(B)*, *bla_KPC-2_*, *bla_GES-2_*, *bla_OXA-2_*, *bla_OXA-48_*, *bla_OXA-10_*, *ant(3″)-Ia*, *ant(3″)-Ii-aac(6′)-IId* (90)	*dfrA14*, *qnrS1*, *sul2*, *mdf(A)*, *mph(B)*, *bla_KPC-2_*, *bla_OXA-48_*, *bla_GES-1_*, *bla_OXA-10_*, *aac(6′)-Ib-cr*, *aph(3″)-Ib*, (79)	*aac(6′)-Ib-cr*, *aadA16*, *bla_KPC-2_*, *sul1*, *qnrB6*, *mdf(A)*, *mph(B)*, *arr-3*, *dfrA27* (82)	*dfrA14*, *sul2*, *bla_OXA-10_*, *bla_GES-1_*, *bla_KPC-2_*, *bla_OXA-48_*, *aph(3″)-Ib*, *mdf(A)*, *qnrS1*, (79)	*sul1*, *tet(A)*, *oqxA*, *oqxB*, *aac(6′)-Ib-cr bla_CTX-M-3_*, *bla_OXA-2_*, *bla_GES-5_*, *bla_KPC-2_*, *fosA*, *aph(3″)-Ib*, *aph(6)-Id*, (62)	*fosA*, *oqxA oqxB*, *bla_GES-5_*, *bla_KPC-2_*, *bla_OXA-2_*, *bla_CTX-M-3_*, *aph(3″)-Ib*, *aph(6)-Id*, *tet(A)*, *sul1* (62)	*oqxB*, *oqxA aac(6′)-Ib-cr*, *sul1*, *fosA*, *bla_KPC-2_*, *bla_GES-5_*, *bla_SHV-11_*, *bla_OXA-48_* (58)	*bla_KPC-2_*, *bla_CMY-157_* (43)
Selected genes linked to virulence (total no.)^a^	*gad*, *lpfA*, *terC*, *yafQ*, *sfaH*, *ldrD*, *potD*, *pemK*, *pemI*, *fliY*, *fliZ*, *fliQ*, *fliA*, *fliG*, *fliI*, *nagA*, *flhA*, *fimD*, *fdeC*, *EspX1*, *epsJ*, *elfG*, *cstA* (30)	*gad*, *lpfA*, *terC*, *sfaH*, *ldrD*, *potD*, *pemK*, *pemI*, *nagA*, *fliY*, *fliZ*, *fliQ*, *fliA*, *fliG*, *fliI*, *flhA*, *fimD*, *EspX1*, *epsJ*, *elfG*, *cstA* (26)	*gad*, *lpfA*, *terC*, *ldrD*, *potD*, *pemK*, *pemI*, *nagA*, *flhA*, *fliY*, *fliZ*, *fliQ*, *fliA*, *fliG*, *fliI*, *fimD*, *EspX1*, *epsJ*, *elfG*, *cstA* (26)	*gad*, *lpfA*, *terC*, *sfaH*, *ldrD*, *potD*, *pemK*, *pemI*, *nagA*, *flhA*, *fliY*, *fliZ*, *fliQ*, *fliA*, *fliG*, *fliI*, *fimD*, *EspX1*, *epsJ*, *elfG*, *cstA* (26)	*sinR*, *sfaG*, *potD*, *fliY*, *fimD*, *feoB eutB* (9)	*sinR*, *sfaG*, *potD*, *fliY*, *fimD*, *feoB*, *eutB* (9)	*sinR*, *sfaG*, *potD*, *pemK*, *pemI*, *fliY*, *fimD*, *feoB*, *eutB*, *cstA* (12)	*potD*, *fliY*, *fliQ*, *fliP*, *fliI*, *fliG*, *fliA*, *flhA*, *eutB*, *cstA* (10)
Plasmids (Inc)	FIB(K), L, P6, C, Col440I, Col(pHAD28)	FIB(K), FII, FII(Yp), L, N, P6, X5, Y, Col440I, Col440II	FIB(K), FII, P6, R, Col440I, Col(pHAD28)	FIB(K), FII, FII(Yp), L, N, P6, Y, Col(IRGK), Col440I, Col440II	FIA(HI1), FII(K), FII(Yp), X5, Y, Col440I, Col440II, Col(pHAD28)	FIA(HI1), FII(K), FII(Yp), X5, Y, Col440I, Col440II, Col(pHAD28)	FIB(K), L, R, Q1, FII(pMET), FII(pKP91), Col440I, Col(pHAD28)	FIB(pHCM2)

a List of total genes associated with the antibiotic resistance and virulence is available in the Supplementary Material.

### *Escherichia coli* M12, M14, M17, and M20 Isolates

The four *bla*_KPC_-carrying *E. coli* isolates subjected to WGS were all of serotype O21:H27 and ST2795. Furthermore, the isolates shared a set of 26–30 virulence-related genes (Table 3), including those encoding the outer membrane usher protein (FimD), flagellar biosynthesis protein (FlhA), and the locus of enterocyte effacement (LEE) encoding the type III secretion system effector protein (EspX1). A number of other genes involved in pathogenicity were discovered as well, including glutamate decarboxylase (*gad*), long polar fimbriae (*lpfA*), tellurium resistance protein (*terC*), toxin-antitoxin systems (*yafQ*, *pemK*, *pemI*), type 1 fimbriae (S - fimbrial adhesion minor subunit; genes *sfaH* and *sfaG*), small toxic polypeptide (*ldrD*), polyamine transport protein D (*potD*), flagellar *fli* genes, laminin-binding fimbriae (*elfG*) and carbon starvation protein A (*cstA*), pointing to the virulence potential of these isolates.

The further similarity between these strains was observed in their plasmid replicon content, with *Inc* replicons of plasmids FIB(K) and P6 detected in all four strains. Nevertheless, each strain exhibited a unique plasmid replicon pattern, comprising 6–10 replicon types per genome (Table 3). More diversity was observed among the ARGs, of which strains M12, M14, M17, and M20 possessed a total of 90, 79, 82, and 79 genes associated with the regulation or acquisition of antibiotic resistance (Table 3; Supplemental Table S1). Nine *bla* genes were identified, including the carbapenemase encoding genes *bla*_KPC-2_ and *bla*_OXA-48_, and ESBL genes *bla*_GES-1_, *bla*_GES-2_, *bla*_OXA-2_, *bla*_OXA-10_, and *bla*_CTX-M-3_. Three strains (M12, M14 and M20) co-harbored *bla*_KPC-2_ and *bla*_OXA-48_. Among others, ARGs mediating resistance to trimethoprim (*dfrA14*), quinolones (*qnrVC4*, *qnrS1*, *qnrB6*), aminoglycosides (*ant(3″)-Ii-aac(6′)-IId*, *ant(3″)-Ia*, *aph(3″)-Ib*) and sulphonamide (*sul1*, *sul2*) were continuously detected.

Moreover, further analysis of the genomes identified the *bla*_KPC-2_ gene in IncP6 plasmid contigs of 38,767, 14,644, 25,016, and 14,644 bp in *E. coli* M12, M14, M17, and M20, respectively.

**Figure 2:**
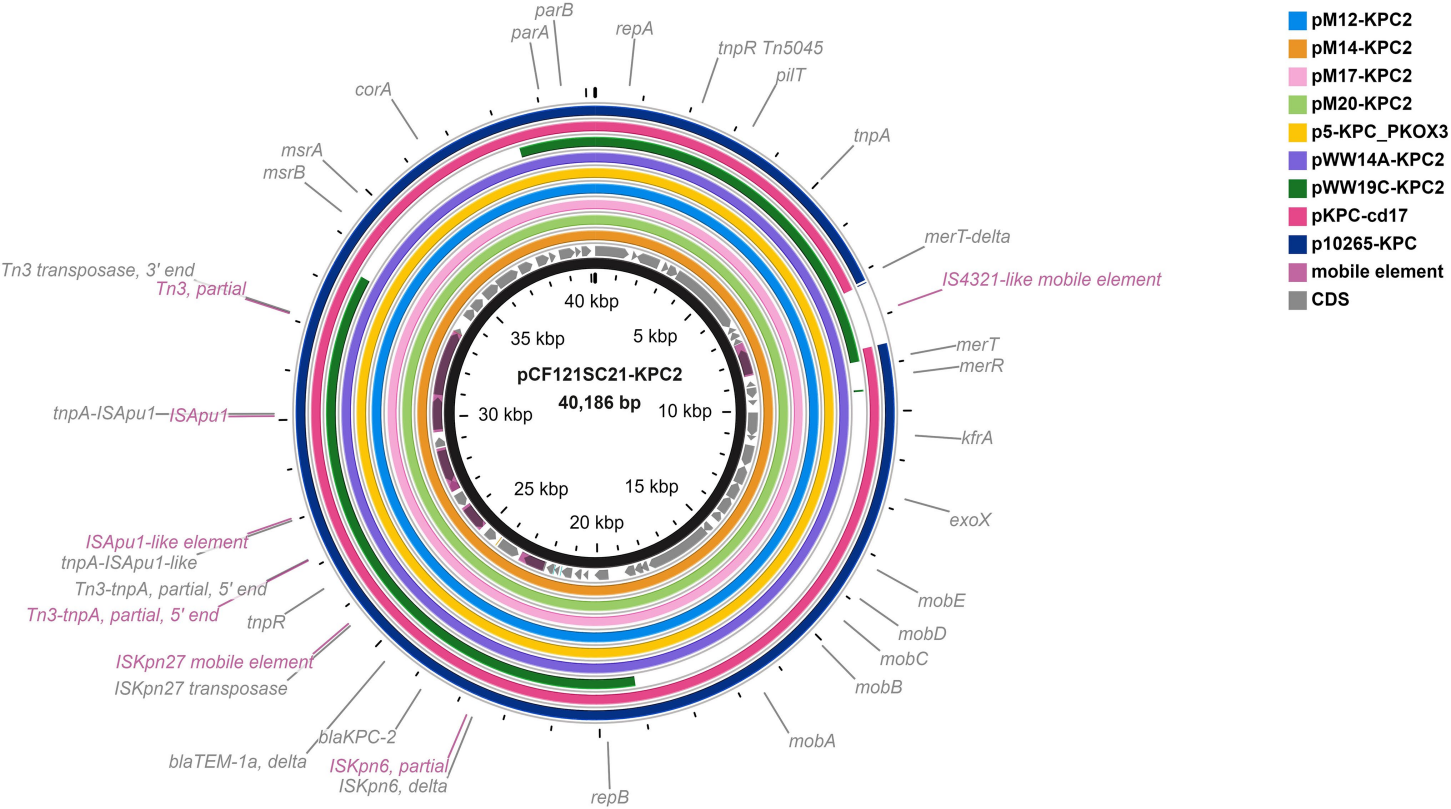
Comparison of plasmids pM12-KPC2, pM14-KPC2, pM17-KPC2, and pM20-KPC2 with IncP6 *bla*_KPC-2_-bearing plasmids of environmental and clinical origin described in the literature: pCF121SC21-KPC2 from Spanish wastewater *Citrobacter freundii* (Genbank accession no. LT992437; Yao et al., 2017), pKOX3-P5-KPC from clinical *Klebsiella oxytoca* in China (GenBank accession no. KY913901; Wang et al., 2017), pWW14A-KPC2 from wastewater *Klebsiella quasipneumoniae* in Argentina (GenBank accession no. CP080103; Ghiglione et al., 2021), pWW19C-KPC2 from wastewater *Enterobacter asburiae* in Argentina (GenBank accession no. CP080110; Ghiglione et al., 2021), p10265-KPC from clinical *Pseudomonas aeruginosa* in China (GenBank accession no. KU578314; Dai et al., 2016), and pKPC-cd17 from *Aeromonas* sp. from hospital environment in United States (GenBank accession no. CP026224) which is 100% identical to the *bla*_KPC-2_-carrying plasmid in *Klebsiella oxytoca* from Spanish hospitals (Pérez-Vazquez et al., 2019). The plasmid pCF121SC21-KPC2 was taken as a reference plasmid (black circle).

BlastN search against NCBI nr database revealed that a 38,767-bp contig from *E. coli* M12 had query coverage of 91% and nucleotide identity of 99.75%, 99.75%, and 99.68% with IncP6 plasmids deposited in GenBank: p121SC21-KPC2 from Spanish wastewater *C. freundii* (Genbank accession no. LT992437; Yao et al., 2017), pKOX3-P5- KPC from a clinical *Klebsiella oxytoca* in China (GenBank accession no. KY913901; Wang et al., 2017), and pWW14A-KPC2 from wastewater *Klebsiella quasipneumoniae* in Argentina (Ghiglione et al., 2021). Hybrid plasmids pM12-KPC2, pM14-KPC2, pM17-KPC2, and pM20-KPC2 were reconstructed to a size of ~40 kb and compared to plasmids of both environmental and clinical origin previously reported in the literature (Dai et al., 2016; Wang et al., 2017; Yao et al., 2017; Pérez-Vazquez et al., 2019; Ghiglione et al., 2021; Figure 2). Analysis of the genetic environment of *bla*_KPC-2_ revealed that this gene is located within a ΔIS*Kpn6*/*bla*_KPC-2_-Δ*bla*_TEM-1_-IS*Kpn27* sequence within a Tn3-based transposon interrupted by an ISApu-flanked element (Figure 2), consistent with previous reports (Dai et al., 2016; Ghiglione et al., 2021).

The *bla*_OXA-48_ was associated with IncL-like plasmids in *E. coli*, but the hybrid *bla*_OXA-48_-bearing plasmids could not be reconstituted (the contigs containing this carbapenemase gene were 2,231-bp long in all four OXA-48-positive *E. coli* and *K. pneumoniae* isolates). However, the IS1R element flanking the OXA-48-encoding gene was detected in *E. coli* M12 isolate.

### *Klebsiella pneumoniae* 5a, M11 and C2 Isolates

Three KPC-2-producing *K. pneumoniae* isolates concurrently carried a total of 62, 58, and 62 genes mediating resistance to multiple antibiotics, respectively (Table 3; Supplemental Table S1). Isolates 5a and M11 were affiliated to ST37, while C2 belonged to ST534. Moreover, isolates 5a and M11 exhibited the same antibiogram and XDR phenotype (Table 1), and a similar ARGs content (Table 3; Supplemental Table S1). However, ERIC and BOX-PCR typing excluded their clonality, and WGS data analysis revealed that these differed by the *aac(6′)-Ib-cr* gene, conferring fluoroquinolone and aminoglycoside resistance, which was found in the genome of strain 5a and not M11. On the other hand, *K. pneumoniae* C2 harbored less ARGs than the former two strains but was resistant to 13 out of 14 tested antimicrobial drugs, remaining susceptible only to trimethoprim-sulfamethoxazole. Nevertheless, identification of *sul1* gene in its genome could eventually result in nonsusceptibility even to this antibiotic. Unfortunately, we were not able to reconstruct *bla*_KPC-2_-and *bla*_OXA-48-_bearing plasmids in *K. pneumoniae* isolates due to the short-read genome sequences.

Moreover, some virulence-related genes have been concurrently detected in all three *K. pneumoniae* genomes (Table 3), codifying for type 1 fimbriae (*fimD*, *sfaG*), ferrous ion transport (*feoB*), ethanolamine-ammonia lyase (*eutB*), extracellular matrix production (*sinR*), flagellae (*fliY*), polyamine transport (*potD*), and nickel and cobalt resistance (*cnrA*), respectively. The C2 strain harbored additional three virulence genes encoding the toxin-antitoxin system (*pemK*, *pemI*) and carbon starvation protein A (*cstA*; Table 3).

### *Citrobacter freundii* CF1

KPC-2-producing *C. freundii* CF1 belonged to ST128 (Table 3). Compared to *E. coli* and *K. pneumoniae* isolates, this strain showed the least diversity of virulence markers, mainly harboring the genes encoding the flagellar apparatus (*fli*), polyamine transport protein D (*potD*), ethanolamine ammonia lyase (*eutB*) and carbon starvation protein A (*cstA*; Table 3). Furthermore, this strain contained the fewest ARGs, 43 in total (Table 3; Supplemental Table S1), and was sensitive to all tested antimicrobial agents (Table 1).

## Discussion

### Carbapenemase-Producing *Escherichia coli* Isolates

All four *bla_KPC_*-carrying *E. coli* isolates that were subjected to WGS belonged to the serotype O21:H27 and ST2795, which was previously identified in the United Kingdom.^5^ The strains carried several genes involved in pathogenicity or other function. For instance, we identified genes encoding the outer membrane usher protein FimD and flagellar biosynthesis protein FlhA associated with the urinary pathogenic *E. coli* (UPEC), and the effector protein EspX1 of the type III secretion system common to enterohemorrhagic *E. coli* (EHEC), all of which were previously detected in wastewater from WWTPs (Zhi et al., 2019). Furthermore, *gad* gene, also detected in all four *E. coli* genomes, is commonly involved in resistance to gastric acid, allowing *E. coli* to survive in the acidic host environment (Mates et al., 2007). The *lpfA* gene, encoding for the long polar fimbriae, was found to be associated with the gut colonization and the attachment to Peyer’s patches in mice (Cordonnier et al., 2017), and was identified in adherent-invasive *E. coli* enrolled in the pathogenesis of Crohn’s disease (Chassaing et al., 2011). Both genes were also identified in the KPC-producing *E. coli* from the riverine environments (Bleichenbacher et al., 2020). Moreover, *terC* gene, encoding the heavy metal resistance, is found to be significantly correlated with the presence of other virulence factors in the pathogenic strains of *E. coli* isolated from humans, animals, and food (Orth et al., 2006).

The PemK/pemI type II toxin-antitoxin system was also detected in the genomes of *bla*_KPC_-positive *E. coli* isolates from this study. This module is consisted of a stable toxin and an unstable antitoxin that degrades under stress conditions, enabling the toxin to inhibit the basic cellular processes. Notably, it has been associated with the bacterial persistence in inhospitable conditions, phage inhibition and biofilm formation (Ramage et al., 2009; Hernandez-Ramirez et al., 2017), as well as the IncF plasmid maintenance, conjugation, and spreading (Walling and Butler, 2016; Diaz-Orejas et al., 2017). This system was previously identified in hypermucoviscous carbapenem-resistant *E. coli* within and outside the hospital environment (Woodford et al., 2009; Mathers et al., 2015; Zurfluh et al., 2018).

Moreover, total of nine carbapenem-resistant *E. coli* isolates from this study were found to harbor *bla*_KPC-2_ gene. It is important to note that the environmental *E. coli* carrying this carbapenemase gene have rarely been reported, and they have all been recovered from the river water (Poirel et al., 2012; Xu et al., 2015; Yang et al., 2017). To the best of our knowledge, this is the first identification of KPC-producing *E. coli* in coastal marine waters. More importantly, the co-occurrence of *bla*_KPC-2_ and *bla*_OXA-48_ in *E. coli* has not been previously reported in the literature. So far, the *bla*_KPC_ gene in *E. coli* has been mainly reported in countries with a high prevalence of KPC-producing *K. pneumoniae*, indicating the possibility of interspecies gene transfer with *K. pneumoniae* serving as a *bla*_KPC_ reservoir (Grundmann et al., 2017). In this regard, we should take into consideration a high prevalence of KPC-producing *K. pneumoniae* in University Hospital Split (Bedenić et al., 2021) and the fact that the isolates were recovered from the water samples collected near the submarine outfall of the WWTP that treats the hospital wastewater. Nevertheless, this presumption should be carefully addressed in future research, focusing on the genetic environment of the hospital KPC-producing strains. To the best of our knowledge, there are no available data on KPC-producing *E. coli* or *K. pneumoniae* from the University Hospital Split analyzed by WGS. The molecular characterization of plasmids harbouring *bla*_KPC-2_ gene in Croatia was performed for clinical *K. pneumoniae*, including those from University Hospital Split that were found to carry this gene on IncFII plasmids (Jelić et al., 2016; Bedenić et al., 2021), or those untyped by PCR-based replicon typing (PBRT; D’Onofrio et al., 2020), as well as in case of river *K. pneumoniae* that harboured *bla*_KPC-2_ gene on IncFII plasmids (Jelić et al., 2019). Notably, *bla*_KPC-2 −_bearing IncP6 plasmids were not previously reported in Croatia.

Moreover, all four *bla*_KPC-2 −_bearing plasmids from *E. coli* isolates in this study were of ~40 kb and highly similar to the IncP6 *bla*_KPC-2_-containing plasmids from wastewater *C. freundii* in Spain (Yao et al., 2017), clinical *K. oxytoca* in China (Wang et al., 2017) and wastewater *K. quasipneumoniae* in Argentina (Ghiglione et al., 2021), pointing to their global circulation. In comparison to plasmids from other incompatibility (Inc) groups, the KPC-2-encoding gene has been rarely detected in IncP6 resistance plasmids (Yao et al., 2017). However, recent studies confirmed that emergence of *bla*_KPC-2_ gene on mobilizable IncP6 broad-host-range plasmids enhanced its dissemination among different members of *Enterobacteriaceae* in clinical settings and the environment (Pérez-Vazquez et al., 2019; Ghiglione et al., 2021). This study provides further evidence to this speculation, documenting for the first time the *bla*_KPC-2_ association with IncP6 plasmids in *E. coli* in Europe.

Furthermore, 6–10 plasmid replicons were detected in *E. coli* genomes. Among them, IncN and Col-type replicons have been previously associated with the occurrence of *bla*_KPC_ in human *E. coli* from the global surveillance studies (Stoesser et al., 2017). In addition, three out of four analysed *E. coli* genomes contained the *bla*_OXA-48_ carbapenemase gene associated with IncL plasmids. Notably, OXA-48-producing *Enterobacteriaceae* have widely disseminated in Croatian hospitals over the past years (Bedenić et al., 2018), with OXA-48-positive *K. pneumoniae* reported at different wards in University Hospital Split. Since IncL-like plasmids were found to enable the transferability of *bla_OXA-48_* in *E. coli* strains from northern Croatia (Bedenić et al., 2018; Drenjančević et al., 2019), findings from this study further enhance their relevance as reservoirs of *bla_OXA-48_* in Croatia.

Moreover, Ambler class A GES-type ESBLs which were identified in KPC-2-producing *E. coli*, including GES-1 (isolates M14 and M20) and GES-2 (isolate M12) may have additionally enhanced their nonsusceptibility to beta-lactams as these enzymes effectively hydrolyse penicillins and expanded-spectrum cephalosporins (Castanheira et al., 2021). GES-2, in comparison to GES-1, also displayes hydrolytic activity against imipenem (Poirel et al., 2001).

In addition, other resistance determinants identified in the genome of our *E. coli* isolates, including the aminoglycoside resistance gene *aph(3″)-Ib*, trimethoprim resistance gene *drfA17* and sulfonamide resistance gene *sul2*, were previously identified in clinical *E. coli* from Croatia (Bedenić et al., 2018).

### Carbapenemase-Producing *Klebsiella pneumoniae* Isolates

In this study, we found that two *bla*_KPC-2_-positive isolates (5a and M11) belong to ST37. Notably, MDR *K. pneumoniae* strains of the same lineage, bearing the *bla_KPC-2_* (Bedenić et al., 2012) and *bla_OXA-48_* (Jelić et al., 2018) carbapenemase genes were previously reported in Croatian hospitals, but not in the natural environment. KPC-producing *K. pneumoniae* of other STs were previously isolated from aquatic environments (Ekwanzala et al., 2019), including ST258 in river water in Croatia (Jelić et al., 2019). Therefore, this study reports the first identification of KPC-producing *K. pneumoniae* in the marine environment in Croatia. Both of *K. pneumoniae* ST37 isolates in this study harbored replicons of plasmids known to enable the spread of ARGs in *Enterobacteriaceae*. Namely, multiple IncF replicons (FIIK, FIB, FIA, and/or FII) were previously identified in *K. pneumoniae* and other *Enterobacteriaceae* (Carattoli, 2009; Huang et al., 2012). On the other hand, *K. pneumoniae* strain C2 was affiliated to ST534, which was previously detected in the hospital environment in Israel (Adler et al., 2015). Our strain additionally harbored IncL and IncR-type plasmid replicons which have been previously described as vehicles of *bla_KPC-2_* (Garbari et al., 2015) as well as *bla_OXA-48_* in Croatia (Bedenić et al., 2018; Drenjančević et al., 2019).

*K. pneumoniae* 51, M11, and C2 isolates produced the Ambler class A GES-5 variant, which confers low carbapenemase activity in addition to penicillins and cephalosporins (Gomi et al., 2018; Castanheira et al., 2021), although GES-5-positive isolates with elevated MICs for imipenem, meropenem, and ertapenem have also been reported (Literacka et al., 2020). In this regard, production of GES-5 may have increased the resistance to carbapenems and other beta-lactams in our isolates. It should be noted that the epidemiology of GES producers is poorly understood, as GES carbapenemase-producing *Enterobacteriaceae* often stay unreported by resulting falsely negative in the Carba NP test due to the relatively weak activity toward carbapenems (Gomi et al., 2018; Literacka et al., 2020). Nevertheless, hospital outbreaks due to carbapenem-resistant GES-5-positive *K. pneumoniae* have recently been reported in Portugal (Mendes et al., 2022) and Poland (Literacka et al., 2020), highlighting their clinical relevance. Apart from the carbapenemases, changes in membrane permeability and activity of membrane efflux pumps may have also contributed to carbapenem resistance in *K. pneumoniae* isolates from this study. Namely, it was observed that a mutant *ompK36* porin gene, like the one detected in these three carbapenemase-producing *K. pneumoniae* isolates, increases nonsusceptibility to this group of antibiotics (Wong et al., 2019). In addition, 7 mutations (P161R, G164A, F172S, R173G, L195V, F197I, and K201M) detected in transcriptional regulator gene *acrR* in C2 isolate were previously shown to highly increase the expression of a major multidrug efflux pump AcrAB-TolC (Sato et al., 2020) that effectively extrude multiple antimicrobials among which carbapenems (Chetri et al., 2019).

Moreover, the environmental *K. pneumoniae* isolates from our study shared similar determinants of resistance to other classes of antibiotics, which were previously described in *K. pneumoniae* clinical isolates from Croatia (Bedenić et al., 2018), such as the aminoglycoside and fluoroquinolone resistance gene *aac(6’)Ib-cr*, disinfectant resistance genes *oqxA* and *oqxB*, sulfonamide resistance gene *sul1*, *fosA* encoding fosfomycin resistance and ESBL gene *bla_CTX-M_*. Moreover, an amino acid substitution R256G was detected in PmrB protein sequence in 5a and M11 strains, which has been previously associated with colistin resistance in *Enterobacteriaceae* (Cheng et al., 2015), as well as in case of CL-resistant and carbapenemase-producing hospital *K. pneumoniae* in Croatia (D’Onofrio et al., 2020). Namely, variations in the PmrB protein, which is a part of the two-component regulatory system PmrA/PmrB enrolled in modification of lipopolysaccharide (LPS) structure, lead to the neutralization of its negative charge and consequently, the reduced susceptibility to cationic peptide antibiotics such as CL. However, as no increased MIC for CL was observed in these isolates, we speculate that the combined action of multiple mechanisms is likely needed to induce resistance to this antibiotic (Cheng et al., 2015). On the other hand, *K. pneumoniae* C2 was resistant to CL (MIC 32 μg/ml), yet no variations in PmrB were found. Limited number of studies have pointed to the underestimated role of the energy-driven efflux pump of peptide antibiotics in *K. pneumoniae*, involving two pumps, AcrAB-TolC and KpnEF (Binsker et al., 2021). It was observed that AcrR deficient mutant strains can successfully extrude polymyxin B, another peptide antibiotic, out of the cell using AcrAB-TolC pump (Padilla et al., 2010). More recent study of Naha et al. (2020) revealed that nonmutated and increasingly expressed RamA, a positive regulator of AcrAB-TolC pump, mediates alterations of LPS which along with the upregulation of the pump have contributed to the CL-resistant phenotype in clinical *K. pneumoniae*. RamA-mediated changes of lipid A moiety have been previously shown to decrease susceptibility to CL in this pathogen (De Majumdar et al., 2015). Therefore, it is likely that a functional AcrAB-TolC system and RamA could also be involved in nonsuceptibility to CL in *K. pneumoniae* C2, but this should be addressed more carefully in the future research.

Moreover, several common virulence-related genes were simultaneously detected in all three *K. pneumoniae* genomes, among which those coding the type 1 fimbriae (*fimD*, *sfaG*) and ferrous ion transport (*feoB*). According to Struve et al. (2008) type 1 fimbriae are significantly enrolled in *K. pneumoniae* infections of urinary tract. The FeoB is the component of the major prokaryotic ferrous ion transport (Feo) system, and the main protein enabling the iron uptake through the lipid bilayer in almost all bacteria (Cartron et al., 2006; Lau et al., 2007). Nevertheless, in this study it was only detected in *K. pneumoniae*, which could be explained by the fact that a single species can adjust its iron import depending on the type of infection (acute or chronic) and the availability of iron in its environment (Cornelis and Dingemans, 2013). On the other hand, the C2 isolate, like *E. coli*, additionally harbored two virulence genes (*pemK*, and *pemI*) encoding the PemK/PemI type II toxin-antitoxin system that has been previously described in hypermucoviscous carbapenem-resistant *K. pneumoniae* (Fu et al., 2018; Bleriot et al., 2020).

### *Citrobacter freundii* CF1 Isolate

*C. freundii* CF1 isolate was affiliated to ST128, which was first described by Bonnin et al. (2020) in an isolate from the rectal swab of a French patient. To date, KPC-producing *Citrobacter* spp. have been isolated from hospital effluents (Zhang et al., 2012), river sediments (Xu et al., 2018), and the recreational areas (Montezzi et al., 2015), but none of them belonged to ST128. To the best of our knowledge, KPC-2-producing *C. freundii* of the ST128 lineage has not been previously reported. It was unexpected that this carbapenem-sensitive isolate resulted positive by CarbaNP test. However, although rare, there are previous reports of KPC-producing Enterobacterales showing unusual carbapenems susceptibility profile while testing positive by CarbaNP (Shinde et al., 2017; Cury et al., 2020), suggesting low gene expression.

Furthermore, we found that the strain CF1 also carried the *bla*_CMY-159_, a variant gene identified only recently (Piotrowska et al., 2019) that encodes for the eponymous AmpC beta-lactamase of the CMY family intrinsic to *Citrobacter* spp. In a later study, this *bla* gene was detected in a *Citrobacter* sp. isolate resistant to cefotaxime, ceftazidime, cefepime and aztreonam, which is similar to the CF1 susceptibility profile. Overall, the majority of the 43 gene markers involved in the antibiotic resistance in this strain was associated with the activity of intrinsic antibiotic efflux including ATP-binding cassette (ABC), RND or MFS pumps. Notably, missense mutations of the AcrAB-TolC efflux pump regulators marR (Y137H) and soxR (T38S) which were detected in this strain were previously found to increase pump expression, leading to the multidrug resistance, among which to beta-lactams and ciprofloxacin (Al-Farsi et al., 2020). Giving the beta-lactam resistance profile of the CF1 isolate (sensitive to carbapenems and cefepime, but resistant to aztreonam and third generation cephalosporins) we can speculate that the mutation-driven expression of the AcrAB-TolC pump did not influence the activity against carbapenems. This would be a case when coincided with the membrane permeability defects resulted from porin loss or porin structural changes (Pages et al., 2008; Vardakas et al., 2012; Sadeghi, 2019), which have not been detected in CF1 isolate.

Moreover, among the 10 virulence-related genes detected in CF1 genome, the major virulence factors of this pathogen such as Shiga-like and heat-stable toxins, or the cholera toxin B subunit homolog (Bai et al., 2012) were not identified, thus we can speculate about the low virulence potential of this isolate (Pepperell et al., 2002).

## Conclusion

This study reports the introduction of XDR and carbapenemase-producing potentially virulent strains of *Enterobacteriaceae* into the Croatian marine environment through the submarine outfall of the treated wastewater located at a depth of 42 m. Among other antibiotic resistance and virulence determinants previously assigned exclusively to clinical strains, we report for the first time KPC-producing *E. coli* in coastal waters and the co-occurrence of *bla_KPC-2_* and *bla_OXA-48_* carbapenemase genes in this species. While *bla*_OXA-48_ was located on an IncL-type plasmids in this species, *bla*_KPC-2_ was harbored by recently described broad-host-range IncP6 resistance plasmids, providing first record of their circulation in *E. coli* and highlighting their importance in the epidemiology of this globally disseminated carbapenemase encoding gene. Leakage of these highly resistant strains into coastal waters through the submarine outlet is of serious concern as it provides a route for their continuous introduction into the marine environment and a reservoir for their further spread.

## Data Availability Statement

The genomic sequences are deposited in the NCBI Sequence Read Archive (BioProject number PRJNA768347) under accession numbers listed in Table 2. The sequence of plasmid pM12-KPC2 from *E. coli* was deposited in GenBank under accession number CP093216.

## Author Contributions

MK and AM: conceptualization and writing—original draft preparation. MDŽ and IŠ: formal analysis. AN, AM, IGB, and MT: validation. MK and AN: investigation. IŠ, JK and AM: resources and funding acquisition. AM: data curation, visualization, and supervision. All authors writing--review and editing and contributed to the article and approved the submitted version.

## Funding

This research was funded by the Croatian Science Foundation (grant number UIP-2019-04-9778), project STIM-REI (KK.01.1.1.01.0003) through the European Regional Development Fund—the Operational Programme Competitiveness and Cohesion 2014–2020 (KK.01.1.1.01), Croatian Academy of Sciences and Arts, and the annual funds for institutional financing of scientific activity from Ministry of Science and Education of Republic of Croatia. The project CAAT “Coastal Auto-purification Assessment Technology” funded by European Union from European Structural and Investment Funds 2014–2020 (KK.01.1.1.04.0064).

## Conflict of Interest

The authors declare that the research was conducted in the absence of any commercial or financial relationships that could be construed as a potential conflict of interest.

## Publisher’s Note

All claims expressed in this article are solely those of the authors and do not necessarily represent those of their affiliated organizations, or those of the publisher, the editors and the reviewers. Any product that may be evaluated in this article, or claim that may be made by its manufacturer, is not guaranteed or endorsed by the publisher.

## Footnotes

^1^ www.ncbi.nlm.nih.gov

^2^ http://www.genomicepidemiology.org

^3^ https://ares-genetics.cloud

^4^ https://beta.proksee.ca/

^5^ https://enterobase.warwick.ac.uk/

## Supplementary Material

Supplementary Table S1 480 Molecular characteristics of eight whole-genome sequenced KPC-producing isolates.
