# Supplementary material for: Submarine Outfalls of Treated Wastewater Effluents are Sources of Extensively- and Multidrug-Resistant KPC- and OXA-48-Producing Enterobacteriaceae in Coastal Marine Environment
Source: Front Microbiol. 2022 May 6;13:858821. doi: 10.3389/fmicb.2022.858821 (PMC9121779; doi:10.3389/fmicb.2022.858821)
Supplement: Supplementary Table S1 — 480 Molecular characteristics of eight whole-genome sequenced KPC-producing isolates. [file Data_Sheet_1.zip › Figure S1.docx]

Supplementary Material

# Supplementary Figures


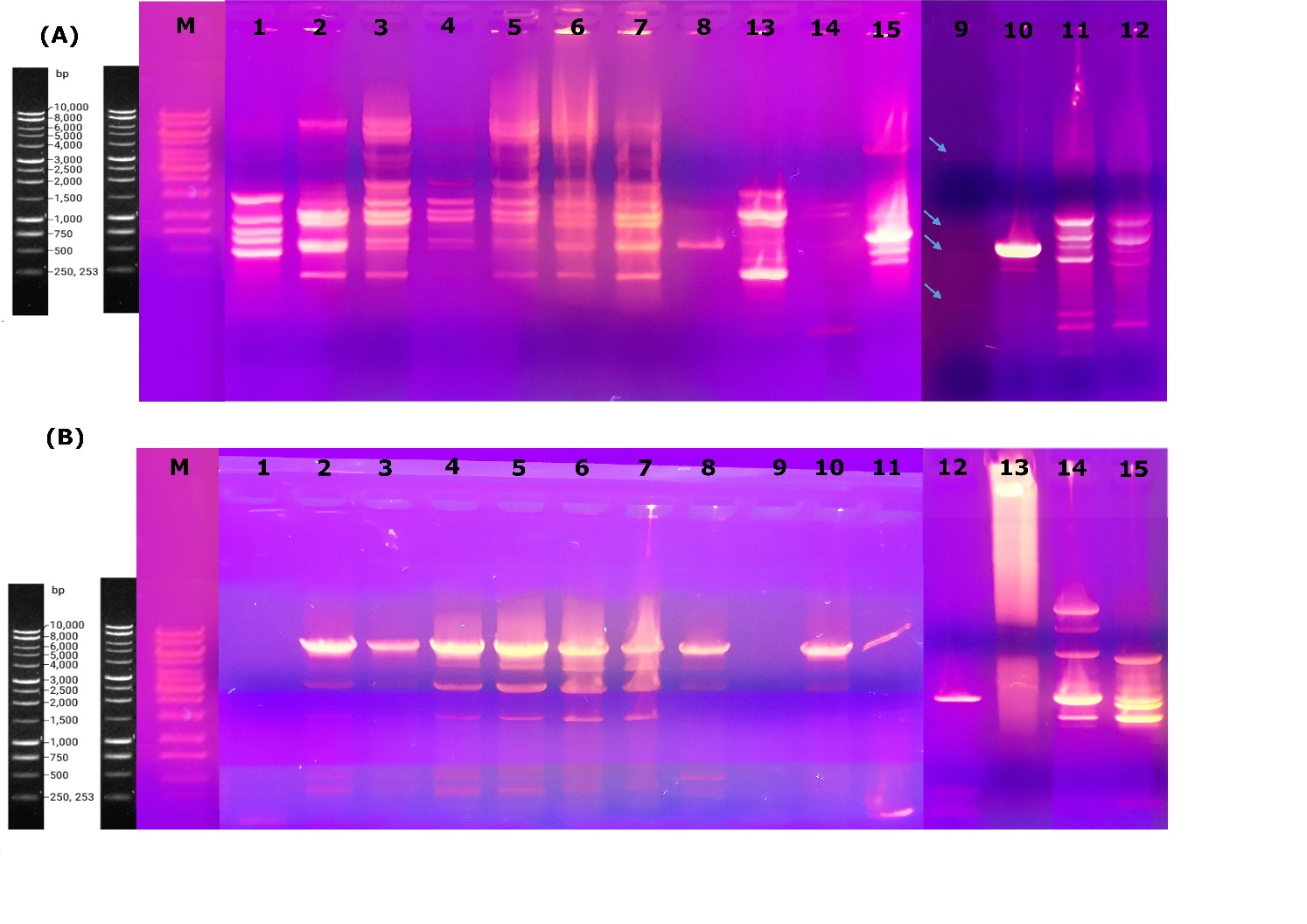


**Supplementary Figure 1.** (A) BOX-PCR and (B) ERIC-PCR profiles of KPC-producing *Enterobacteriaceae*: *E. coli* isolates M20 (line 2), M19 (line 3), M16 (line 4), M13 (line 5), M14 (line 6), M15 (line 7), M17 (line 8), M12 (line 10), M18 (line 13); *K. pneumoniae* isolates 5a (line 1), M11 (line 9), C2 (line 11) and C1 (line 12); *C. freundii* isolates CF1 (line 14) and CF2 (line 15). 1 kb DNA ladder (Promega, USA) was used.
